# Frequency-dependent gating of feedforward inhibition in thalamofrontal synapses

**DOI:** 10.1186/s13041-020-00608-2

**Published:** 2020-05-06

**Authors:** Jungmin Lee, Joon Ho Choi, Jong-Cheol Rah

**Affiliations:** 1 Korea Brain Research Institute, 61 Cheomdan-ro, Daegu, 41062 Republic of Korea; 2 Department of Brain and Cognitive Sciences, Daegu Gyeongbuk Institute of Science & Technology, Daegu, 42988 Republic of Korea

**Keywords:** Thalamofrontal, Feedforward inhibition, Mediodorsal nucleus of the thalamus, Dorsal anterior cingulate cortex, Short-term memory

## Abstract

Thalamic recruitment of feedforward inhibition is known to enhance the fidelity of the receptive field by limiting the temporal window during which cortical neurons integrate excitatory inputs. Feedforward inhibition driven by the mediodorsal nucleus of the thalamus (MD) has been previously observed, but its physiological function and regulation remain unknown. Accumulating evidence suggests that elevated neuronal activity in the prefrontal cortex is required for the short-term storage of information. Furthermore, the elevated neuronal activity is supported by the reciprocal connectivity between the MD and the medial prefrontal cortex (mPFC). Therefore, detailed knowledge about the synaptic connections during high-frequency activity is critical for understanding the mechanism of short-term memory. In this study, we examined how feedforward inhibition of thalamofrontal connectivity is modulated by activity frequency. We observed greater short-term synaptic depression during disynaptic inhibition than in thalamic excitatory synapses during high-frequency activities. The strength of feedforward inhibition became weaker as the stimulation continued, which, in turn, enhanced the range of firing jitter in a frequency-dependent manner. We postulated that this phenomenon was primarily due to the increased failure rate of evoking action potentials in parvalbumin-expressing inhibitory neurons. These findings suggest that the MD-mPFC pathway is dynamically regulated by an excitatory-inhibitory balance in an activity-dependent manner. During low-frequency activities, excessive excitations are inhibited, and firing is restricted to a limited temporal range by the strong feedforward inhibition. However, during high-frequency activities, such as during short-term memory, the activity can be transferred in a broader temporal range due to the decreased feedforward inhibition.

## Introduction

The activity patterns of inhibitory neurons play a critical role in sculpting cortical network dynamics. Thalamic excitatory inputs diverge on both excitatory and inhibitory cortical neurons, generating disynaptic feedforward inhibition. Despite the fact that thalamic efferent inputs on parvalbumin-expressing (PV) interneurons are bifurcated from the same set of axons, their pre- and postsynaptic mechanisms tend to be stronger than those on principal neurons [1–3]. Furthermore, the connection probability of GABAergic interneurons is remarkably higher than that of pyramidal neurons [4–7]. Thereby, feedforward inhibition dominates the excitatory responses and limits the temporal window for integration of excitatory thalamic inputs (hereafter referred to as the “integration window”) [8, 9]. It is well accepted that feedforward inhibition sharpens the spatial and temporal discrimination of sensory information [2, 10, 11]. In the medial prefrontal cortex (mPFC), the mediodorsal thalamic nucleus (MD) drives feedforward inhibition in the dorsal anterior cingulate cortex (dACC) via local parvalbumin-expressing GABAergic neurons [12].

The increased and sustained neural activity of the mPFC has been widely believed to be the substrate of short-term memory [13, 14]. Studies in monkeys and rodents have demonstrated that functional interaction with the reciprocally connected MD is critical for maintaining working memory [15–17]. Specifically, interrupting this interaction caused coincident increases in firing in the MD, reduced reverberant activity in the prefrontal cortex (PFC), and reduced performance during short-term memory-dependent tasks [13, 15, 16, 18]. In many of these experiments, recorded units in the mPFC as well as in the MD during the delay period of the tasks exhibited high-frequency firing, often over 10 Hz [17, 19, 20].

Although feedforward inhibition mediated by cortical PV neurons has been described previously [12], the mechanism underlying the modulation of feedforward inhibition during high-frequency activity has never been examined. In this study, we examined how feedforward inhibition in MD-dACC connectivity is modulated during high-frequency activity with whole-cell recordings with optogenetic stimulation to better understand how feedforward inhibition is modulated during short-term memory.

## Methods

### Animals

Genetically modified mouse lines were purchased from Jackson Laboratories and bred in-house. The PV-Cre mouse line (B6;129P2-*Pval*^*btm1(cre)Arbr*^/J; JAX stock #008069) was used to target PV-expressing interneurons. The PV-Cre mouse line was bred with Ai9 mice (B6.Cg-*Gt (ROSA)26Sor*^*tm9(CAG-tdTomato)Hze*^/J; JAX stock #007909) to identify PV neurons. PV interneurons of the PV:Cre/Ai9 line express robust tdTomato fluorescence following Cre-mediated recombination. To selectively evoke APs in PV neurons, PV-cre mice were bred with Ai27D mice (B6.Cg-*Gt [ROSA]26Sor*^tm27.1[CAG-COP4*H134R/tdTomato]Hze^/J; JAX stock #012567). PV neurons of the PV-Cre/Ai27D line express the improved channelrhodopsin-2/tdTomato fusion protein. The PV-Cre, Ai27D, and Ai9 transgenic lines were bred as homozygotes. Mice were housed under a 12-h light-dark cycle with ad libitum access to food and water. Only male mice were used to avoid the potential effect of the estrus cycle. All care procedures involving animals were approved by the Institutional Animal Care and Use Committee of the Korea Brain Research Institute (M2-IACUC-19-00040).

### Viral vectors and stereotactic surgeries

Animals were anesthetized with ketamine (100 mg/kg) supplemented with dexmedetomidine hydrochloride (0.4 mg/kg) by intraperitoneal injection and positioned in a stereotaxic injection frame (Kopf instruments). Ketoprofen (5 mg/kg) was subcutaneously injected for its anti-inflammatory effects. During the surgery, responses to pedal withdrawal reflex stimuli were absent. Virus injection was conducted followed by a stereotaxic surgical procedure.

An EF1a-driven, Cre-dependent, humanized channel rhodopsin (hChR2) H134R mutant fused to enhanced yellow fluorescent protein (eYFP) for optogenetic activation (Addgene # 20298-AAV1) and hSyn-Cre (Addgene # 105553-AAV1) was transduced by adeno-associated virus serotype 1 (AAV1). Between postnatal days 40–50, the mixture (50:50) of AAV1-double floxed-H134R and AAV1-Cre was unilaterally injected into the MD of the PV:Cre/Ai9 mouse. To derive PV-induced inhibitory postsynaptic currents (IPSCs) in the PFC, AAV1-double floxed-H134R was bilaterally injected into the *PV-Cre* mouse PFC. Approximately 70 nL of the virus solution (10^12^ viral particles/mL) was delivered with a glass micropipette (Drummond Scientific) through a small skull window (1–2 mm^2^). To avoid leakage into surrounding brain areas, we left the injection pipettes in the brain for 6 min after the injection before slowly withdrawing them. The injections were performed using following stereotaxic coordinates. The MD coordinates from the bregma were as follows: anterior-posterior, − 1.58 mm; medial-lateral, ± 0.30 mm; dorsal-ventral, − 3.10 mm. The PFC coordinates from the bregma were as follows: anterior-posterior, 1.75 mm; medial-lateral, ± 0.30 mm; dorsal-ventral, − 1.00 mm. During all surgical procedures, the animals were kept on a heating pad in an isolated cage and were brought back to their home cages when they regained movement. For optimal viral expression, all mice were euthanized at least 3 weeks after the surgery.

### Electrophysiology and optogenetics

Mice aged 9–10 postnatal weeks were euthanized by exposure to CO_2_ followed by decapitation. The brains were quickly and carefully removed in ice-cold dissection solution: 25 mM sodium bicarbonate (NaHCO_3_), 1.25 mM sodium monophosphate (NaH_2_PO_4_), 25 mM D-glucose, 2.5 mM KCl, 7 mM MgSO_4_·6H_2_O, 0.5 mM CaCl_2_, 110 mM choline chloride, 11.61 mM (+) sodium-L-ascorbate, and 3 mM sodium pyruvate. The measured osmotic concentration was between 320 and 330 mOsm. Acute 300-μm thick brain slices were prepared via coronal sections with a vibratome (Leica VT1200S) in ice-cold dissection solution. The composition of artificial cerebrospinal fluid (aCSF) was as follows: 119 mM NaCl, 2.5 mM KCl, 1 mM MgSO_4_·7H_2_O, 26 mM sodium bicarbonate (NaHCO_3_), 1.25 mM sodium monophosphate (NaH_2_PO_4_), 20 mM D-glucose, 0.4 mM L-ascorbic acid, 2 mM CaCl_2_, and 2 mM pyruvic acid. The measured osmotic concentration was between 305 and 310 mOsm. After 30 min of recovery time in warmed aCSF (32 °C), slices were transferred to room temperature. For each mouse, PFC slices were prepared first, and then slices around the MD were prepared to ensure that slices of the injection sites were obtained. Mice were excluded from data analysis whenever expression of the virus was observed outside of the MD. The dACC L2/3 pyramidal neurons and PV interneurons were recorded either by voltage clamping or current clamping for the following procedures.

PV neurons were discerned visually and electrophysiologically by measurements of intrinsic properties. PV interneurons exhibited higher firing rates with minimal adaptation and a lower AP threshold. All recordings, including voltage holding at − 30 mV, were performed with patch pipettes (3.5–4 MΩ) filled with an internal solution that consisted of the following components: 20 mM KCl, 125 mM K-gluconate, 10 mM HEPES, 4 mM NaCl, 0.5 mM EGTA, 4 mM ATP, 0.3 mM Tris-GTP, and 10 mM phosphocreatine with a pH adjusted to 7.2 with KOH. The measured osmotic concentration was between 307 and 314 mOsm. Recordings were performed at room temperature.

Optogenetic stimulation was applied with a 1-ms light pulse from a 470-nm laser source; the light was guided with an optic fiber placed within 1 mm of the recorded neurons. The total power of the laser measured at the tip of the fiber by Power Meter (Thorlabs) was ~ 5 mW/mm^2^.

We measured the resting potential of all neurons in current-clamp mode immediately after rupture of the neuronal membrane. Series resistance was determined by measuring the voltage change in response to a small hyperpolarizing current pulse (5 pA, 50 ms) at resting potential. Spike threshold was acquired by 20-pA step increments of current injection and determined as the point at which the first AP was evoked. Series resistance was observed throughout the entire experiment and was not compensated. Cells with series resistances over 20 MΩ were excluded.

All solutions were kept saturated with 95% O_2_ and 5% CO_2_. Acute slices were continuously perfused with aCSF at room temperature. All data except the experiment performed with PV-Cre/Ai27D mice were sampled at 20 kHz by the EPC-10 amplifier (HEKA Elektronik) with Patchmaster software (HEKA Elektronik) and further analyzed by MATLAB (Mathworks). Electrophysiological data for the experiment using PV-Cre/Ai27D mice were recorded using an Axopatch 700B amplifier (Molecular Devices), and command pulse generation was performed using Digidata 1550 (Molecular Devices). The data were further analyzed using Clampfit 10.4 (Axon Instruments) and IGOR Pro software (Wavemetrics).

### Drug application

The following drugs were perfused in aCSF: 100 μM AP5 ([2R]-amino-5-phosphonovaleric acid, an N-methyl-D-aspartate (NMDA) receptor antagonist, Tocris; IC_50_ = ~ 50 μM), 10 μM CNQX (6-cyano-7-nitroquinoxaline-2,3-dione, an AMPA/kainate receptor antagonist, Tocris; IC_50_ = 1.5 μM), 2 μM bicuculline (ionotropic γ-aminobutyric acid or GABA_A_ receptor antagonist, Sigma-Aldrich; IC_50_ = 2 μM), 0.5 μM TTX (tetrodotoxin, a Na^+^ channel blocker, Abcam; IC_50_ < 10 nM), and 100 μM 4-AP (4-aminopyridine, a Kv1 channel blocker, Tocris; IC_50_ = 147 μM). All drugs were perfused throughout the experimental protocol and washed out for at least 30 min after the end of the protocol.

### Statistics

Data analysis was performed using MATLAB (Mathworks), and GraphPad Prism 6.0 (GraphPad Software). Data are presented as the mean ± standard deviation unless otherwise noted. Parametric or non-parametric tests were employed according to the normality tests. Statistical analyses were performed using a two-tailed Student’s *t*-test for the comparison of two groups. For comparisons across more than two groups, data were analyzed using one-way analysis of variance followed by Tukey’s post hoc analysis to correct for multiple comparisons. For data with a non-normal distribution, the non-parametric Mann-Whitney or Wilcoxon signed-rank tests were used. A *P* value < 0.05 was considered statistically significant.

## Results

**Fig. 1:**
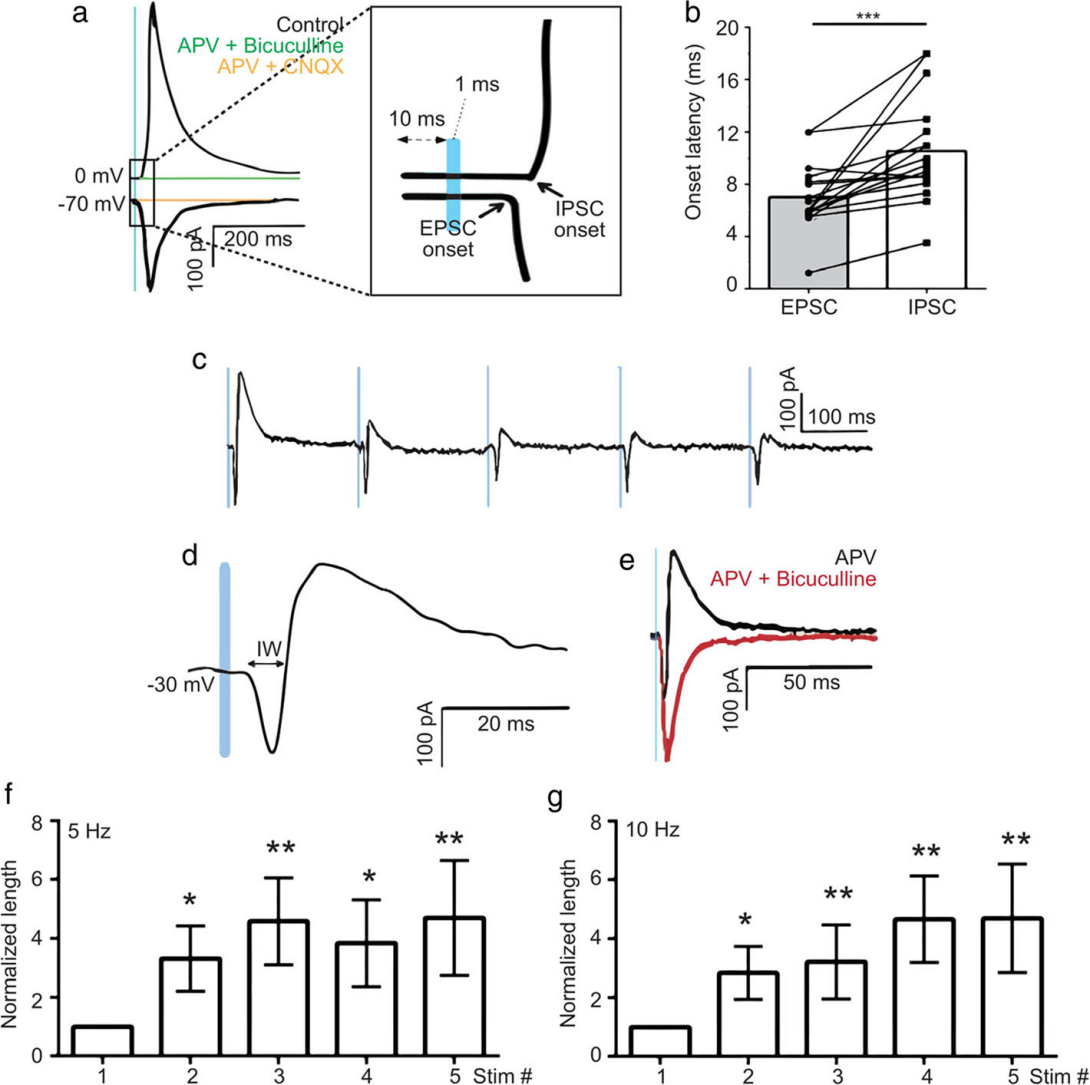
The thalamofrontal integration window increases at high frequency. **a** Representative traces of the synaptic current. An inhibitory postsynaptic current (IPSC) measured at 0 mV (outward black trace) and an IPSC in the presence of a GABA_A_ receptor antagonist (2 μM bicuculline, green trace). An EPSC measured at − 70 mV (inward black trace) and an EPSC in the presence of 10 μM 6-cyano-7-nitroquinoxaline (CNQX; orange trace). An NMDA channel-dependent current was ruled out by 100 μM (2R)-amino-5-phosphonopentanoate (APV) throughout the experiment. Magnified EPSC and IPSC traces around the onset of the synaptic currents **(inset)**. The inflection points of the EPSC and IPSC were defined as the onsets and used to calculate the onset latencies. **b** The onset latency of the thalamofrontal IPSC on pyramidal cells was longer than that of the EPSC (17 cells, ***p = 0.0008, paired *t*-test, parametric). **c** Example trace of the EPSC/IPSC complex sequence at − 30 mV with 5 Hz optogenetic thalamofrontal stimulation. **d** An example trace showing how the integration window was measured, namely, as the duration of the net inward current in EPSC-IPSC sequences. **e** An EPSC-IPSC sequence with and without 2 μM bicuculline (Vhold = − 30 mV, red). **f−g** The normalized length of the integration window at 5 Hz (**f**) (9 cells, *P_stim2_ = 0.023, **P_stim3_ = 0.0039, *P_stim4_ = 0.012, **P_stim5_ = 0.0078, paired *t*-test, non-parametric) and 10 Hz (**g**) (9 cells, *P_stim2_ = 0.016, **P_stim3_ = 0.0078, **P_stim4_ = 0.0039, **P_stim5_ = 0.0078, paired *t*-test, non-parametric)

To selectively examine the thalamofrontal synapses and recruited local inhibitory inputs, we transduced the MD with adeno-associated virus (AAV)-channelrhodopsin-2 (ChR2) (Fig. 1). As previously described [12], optogenetic stimulation delivered to thalamofrontal axons with a 470-nm laser evoked both excitatory and inhibitory synaptic currents on pyramidal neurons in the dACC L2/3. We observed a large depolarizing inward current near the reversal potential of chloride (Fig. 1a). When the membrane voltage (Vm) was clamped near 0 mV, we observed a hyperpolarizing outward current that was sensitive to the GABA_A_ receptor antagonist bicuculline (2 μM), suggesting that the current was an inhibitory synaptic current, i.e., an IPSC (Fig. 1a). The IPSCs were also completely blocked by the AMPA/kainate receptor antagonist 6-cyano-7-nitroquinoxaline (CNQX, 10 μM), indicating that the observed IPSCs were not directly from the MD but from local inhibitory neurons excited by the MD [12]. To further confirm that the IPSCs were current from disynaptically connected interneurons, we compared the onset latency of IPSCs with that of excitatory postsynaptic currents (EPSCs) (Fig. 1a, right). As expected, the onset of the IPSCs appeared significantly delayed (EPSCs, 7.06 ± 0.62 ms; IPSCs, 10.58 ± 0.96 ms; 17 cells, *P* = 0.0008, paired *t*-test; Fig. 1b). The observed delay was 3.52 ± 0.86 ms and corroborated well with the synaptic delays described in previous studies [12, 21]. We concluded that feedforward inhibition was driven by activation of MD axons in the dACC.

We then examined how the temporal window of excitation is modulated by high-frequency MD activity. The neurons were voltage-clamped at − 30 mV, and a series of optogenetic stimulus-evoked biphasic inward and outward currents were observed (Fig. 1c). We defined the integration window as the temporal duration of the net inward current in this condition (Fig. 1d) [2, 22]. The measured integration window upon a low-frequency stimulus was approximately 6.02 ± 0.88 ms (mean ± range). Upon high-frequency (5 Hz or 10 Hz) MD activity, the width of the integration window increased significantly (Fig. 1f−g). The normalized length of the integration window gradually increased as the stimulation continued (Fig. 1f−g). The length of the integration window upon the fifth stimulus was approximately five-fold wider than that of the first window (5 Hz: 6.45 ± 1.37 ms vs. 30.01 ± 13.14 ms, 9 cells; 10 Hz: 5.58 ± 1.18 ms vs. 21.33 ± 8.43 ms, 9 cells, mean ± range).

**Fig. 2:**
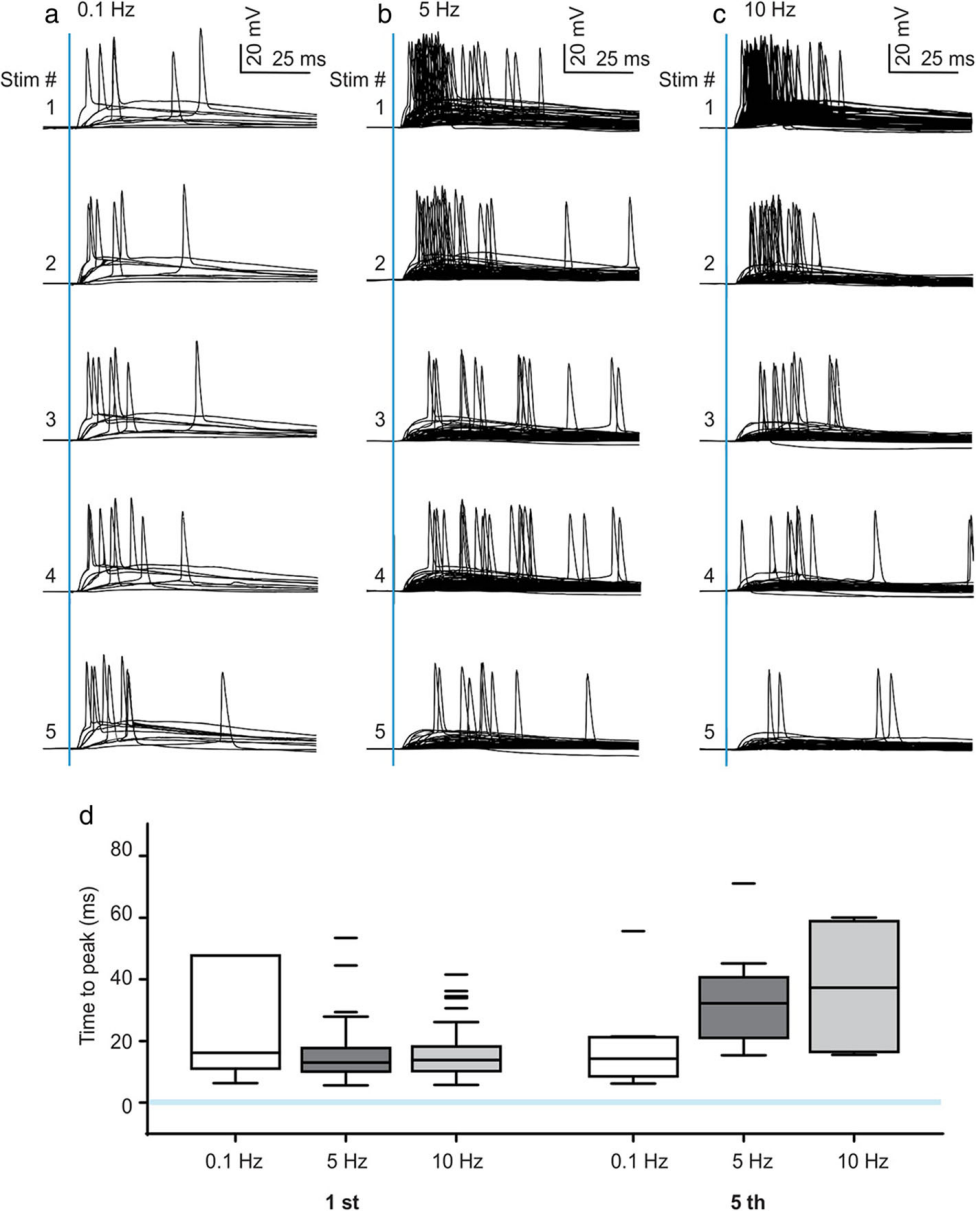
The dorsal anterior cingulate cortex L2/3 pyramidal spike output jitters along with high-frequency stimulation. **a−c** Superimposed traces of voltage changes due to thalamofrontal optogenetic stimulation at **a** 0.1 Hz (9 cells), **b** 5 Hz (53 cells), and **c** 10 Hz (48 cells). Light stimulations (1 ms) are shown as a blue vertical line. **d** The time-to-peak of optogenetically evoked action potentials after the first and fifth stimulations at the indicated frequencies. The stimulation points are shown as a blue horizontal line

Weakened feedforward inhibition during high frequency predicts greater temporal variability of action potential (AP) generation. We measured AP jitter between the stimulus and AP peaks with whole-cell voltage recordings and optogenetic stimulation of MD axons (Fig. 2). As expected by the strong depression of thalamofrontal excitatory inputs, we observed a strongly reduced firing rate and delayed AP generation as high-frequency stimuli proceeded. Furthermore, we observed greater AP jitter during high-frequency activity of MD axons. The firing ranges of the frequencies were initially 22.48 ± 16.42 ms at 0.1 Hz, 17.59 ± 18.47 ms at 5 Hz, and 15.92 ± 8.30 ms at 10 Hz each. After five consecutive stimulations, the ranges became much broader with high-frequency activity (13.96 ± 6.02 ms at 0.1 Hz, 33.66 ± 16.81 ms at 5 Hz, and 37.56 ± 23.38 ms at 10 Hz).

**Fig. 3:**
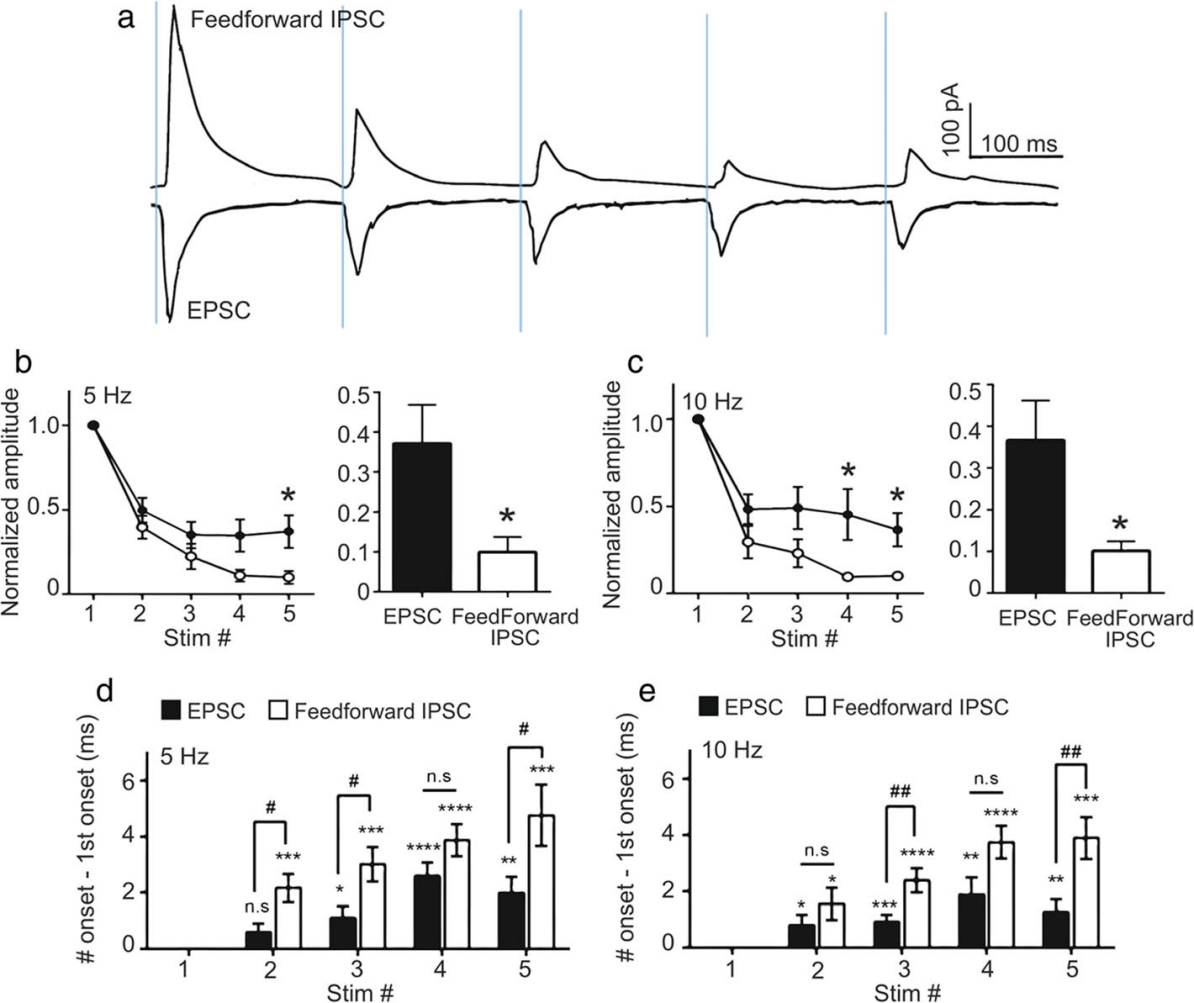
Feedforward inhibitory postsynaptic currents (IPSCs) depress faster than excitatory postsynaptic currents (EPSCs). **a** Sample traces of an EPSC and IPSC in dorsal anterior cingulate cortex (dACC) L2/3 pyramidal neurons. **b−c** Normalized amplitudes of an EPSC and IPSC at 5 Hz (**b**) and 10 Hz (**c**) (7 and 5 cells were recorded for the EPSC and IPSC, respectively; 5 Hz P_stim2_ = 0.35, P_stim3_ = 0.28, P_stim4_ = 0.073, *P_stim5_ = 0.047, unpaired *t*-test, parametric; 10 Hz P_stim2_ = 0.17, P_stim3_ = 0.10, *P_stim4_ = 0.050, *P_stim5_ = 0.032, unpaired *t*-test, parametric). **d−e** Onset latencies of EPSC and IPSC after consecutive stimulations at **d** 5 Hz and **e** 10 Hz (17 and 8 cells were recorded for the EPSC and IPSC, respectively; EPSC 5 Hz, P_stim2_ = 0.080, *P_stim3_ = 0.013, ****P_stim4_ < 0.0001, **P_stim5_ = 0.0011, unpaired *t*-test, parametric; EPSC 10 Hz, *P_stim2_ = 0.40, ***P_stim3_ = 0.0008, **P_stim4_ = 0.0038, **P_stim5_ = 0.0082, unpaired *t*-test, parametric; IPSC 5 Hz, ***P_stim2_ = 0.0007, ***P_stim3_ = 0.0002, ****P_stim4_ < 0.0001, ***P_stim5_ = 0.0007, unpaired *t*-test, parametric; IPSC 10 Hz, *P_stim2_ = 0.015, ****P_stim3_ < 0.0001, ****P_stim4_ < 0.0001, ***P_stim5_ = 0.0001, unpaired *t*-test, parametric; *P* value between EPSC and IPSC groups, 5 Hz, #P_stim2_ = 0.013, #P_stim3_ = 0.017, P_stim4_ = 0.13, #P_stim5_ = 0.020, unpaired *t*-test, parametric; *P* value between EPSC and IPSC groups, 10 Hz, P_stim2_ = 0.26, ##P_stim3_ = 0.0038, P_stim4_ = 0.069, ##P_stim5_ = 0.0044, unpaired *t*-test, parametric)

These results demonstrate that feedforward inhibition, and thereby the integration window of dACC neurons, are dynamically regulated in a frequency-dependent manner. Next, we examined the circuit mechanisms by which the integration window was selectively widened in the presence of high-frequency MD activity. Lengthening of the integration window can occur by the facilitation of EPSCs [23] and/or depression of IPSCs [2]. The first possibility is unlikely because MD-to-mPFC synapses, similar to other thalamocortical synapses, have been shown to have a high probability of neurotransmitter release and tend to be depressed by high-frequency stimulation [24]. Therefore, we focused on the possibility of reduced feedforward inhibition [2, 8]. To test this idea, we compared the changes in thalamofrontal EPSCs and feedforward IPSCs during high-frequency activity (Fig. 3). As previously shown, MD-dACC synapses were depressed strongly during high-frequency activity [24]. However, the indirect inputs via cortical inhibitory neurons showed a remarkably faster short-term depression [25] (Fig. 3b−c). The amplitudes of IPSCs depressed to 0.10 ± 0.04 and 0.10 ± 0.02 of the first amplitude by five consecutive stimuli at 5 and 10 Hz, respectively (5 cells), whereas the amplitudes of EPSCs decreased to 0.37 ± 0.10 and 0.37 ± 0.10 of the initial current by five consecutive stimuli at 5 and 10 Hz, respectively (7 cells).

The reduced feedforward IPSCs accompanied by widened integration windows can be explained by the failure or delayed onset of APs in the inhibitory neurons. In other words, the strong short-term depression of the MD-driven synaptic current during tetanic stimulation could lengthen the time needed or even fail to evoke APs in the cortical inhibitory neurons. We first examined this possibility by comparing the onset of EPSCs and IPSCs during a series of stimulations (Fig. 3d−e). Supporting the idea of delayed AP onsets, we found that the temporal differences between the onsets of the EPSCs and the feedforward IPSCs were more pronounced with consecutive stimulations.

**Fig. 4:**
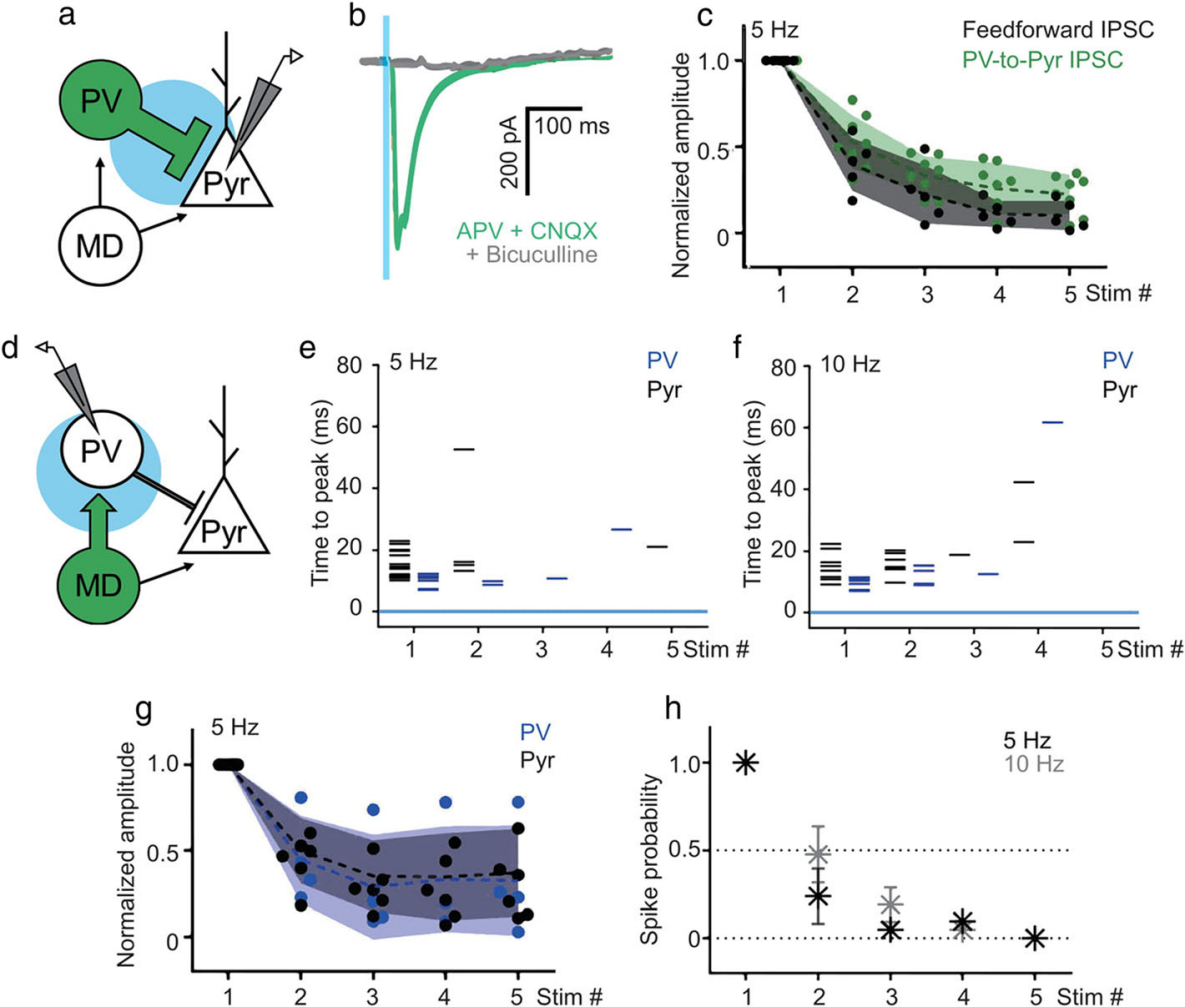
Short-term plasticity of PV-IPSCs and thalamofrontal EPSCs in PV neurons in the dACC L2/3. **a** Schematic image of the experiment. Inhibitory postsynaptic currents (IPSCs) derived from local parvalbumin-expressing (PV) activity were measured in pyramidal neurons in the dorsal anterior cingulate cortex (dACC). **b** An example trace of a PV-derived IPSC in the presence of (2R)-amino-5-phosphonopentanoate (APV) and 6-cyano-7-nitroquinoxaline (CNQX; green) after bicuculline (gray) (Vhold = − 70 mV). Light stimulations (1 ms) are shown as a blue vertical line. **c** PV-IPSCs were depressed similarly to the feedforward disynaptic IPSCs evoked by mediodorsal nucleus of the thalamus stimulation at 5 Hz (8 cells, P_stim2_ = 0.16, P_stim3_ = 0.18, P_stim4_ = 0.07, P_stim5_ = 0.06, unpaired *t*-test, parametric). Standard deviation is depicted as the shaded area. **d** Schematic image of a thalamofrontal EPSC in PV neurons. **e−f** Each single action potential time-to-peak in PV and pyramidal neurons at 5 Hz (**e**) and 10 Hz (**f**) is shown as a horizontal bar (9 trials). **g** Normalized thalamofrontal EPSC amplitude on PV compared with that on pyramidal neurons at 5 Hz (4 PV cells, 6 Pyr cells, P_stim2_ = 0.72, P_stim3_ = 0.69, P_stim4_ = 0.94, P_stim5_ = 0.79, unpaired *t*-test, non-parametric, thalamofrontal EPSCs in pyramidal neurons are in the same data set as that used for Fig. 3b and c). Standard deviation is depicted as the shaded area. **h** Spike probability of PV neurons at 5 Hz and 10 Hz (7 cells, P_stim2_ = 0.13, P_stim3_ = 0.5, P_stim4_ > 0.9, P_stim5_ = 1.0, unpaired *t*-test, non-parametric)

We tested two possibilities to determine the stronger depression of disynaptic feedforward IPSCs. We first examined the short-term plasticity of PV-pyramidal synapses. To selectively evoke APs in cortical PV neurons, we expressed channelrhodopsin-2 on PV neurons either by introducing AAV-double floxed-ChR2-EYFP (Addgene #20298) to PV-Cre mice, or by using PV-ChR2 mice that express hChR2 in PV neurons. Upon 5 Hz tetanic stimulation of PV-pyramidal synapses, synaptic depression of IPSCs of MD-pyramidal synapses was indistinguishable from feedforward inhibition derived from MD stimulation on pyramidal neurons in PV-ChR2 mice (8 cells, 0.22 ± 0.04 of the initial plateau amplitude by the fifth response during 5 Hz stimulation; Fig. 4a−c). Furthermore, short-term depression of IPSCs from PV neurons-to-pyramidal neurons was not greater than that of the thalamofrontal EPSCs (Supplementary Fig. 1A). This result indicates that the short-term synaptic plasticity of PV-pyramidal synapses alone cannot account for the extraordinarily fast synaptic depression of feedforward inhibition.

Previous studies have shown that PV neurons receive greater and more frequent responses from thalamic inputs [1–3] and mediate feedforward inhibition [12, 26]. Therefore, it is probable that MD inputs on PV synapses depress too fast to evoke reliable APs in PV neurons during tetanic stimulation. To test this, we identified and recorded voltage changes specifically from PV neurons during the high-frequency activity of MD synapses in the PV-TdTomato mouse line. In response to high-frequency optogenetic stimulation, the probability of successful AP generation by activation of MD axons dropped dramatically (Fig. 4d-h). However, the short-term plasticity of thalamofrontal EPSCs in PV neurons were not significantly greater than that in pyramidal neurons (Fig. 4g). Of note, although it was not statistically significant, the reduction in the spike probability of PV neurons tended to decay faster than the synaptic input; subsequently, no AP was generated by the fifth stimulus (Fig. 4g, h and Supplementary Fig. 1B). We attributed the dramatic reduction of AP generation to a relatively high rheobase of PV neurons in the cortices [27, 28]. The observed failure in AP generation was not due to ChR2 inactivation, as optogenetic 10 Hz stimulation evoked immediate and reliable APs in ChR2-expressing MD neurons in our experimental condition (data not shown). Therefore, we concluded that, during the high-frequency activity of the MD, thalamofrontal synapses depress rapidly enough to fail in recruiting feedforward inhibition.

## Discussion

The current findings indicate that feedforward inhibition decreases during high-frequency stimulation, and thereby, the integration of excitation can be dynamically regulated during high-frequency MD activity, such as during short-term memory. During high-frequency activity, we observed frequent failure in excitation transfer from MD to PV neurons in the dACC and, in turn, rapidly decreased feedforward inhibition.

The temporal window of integration was widened accordingly, which increased the probability of thalamofrontal excitatory inputs being integrated. Frequency-dependent gating of the feedforward inhibition, and thus regulation of the integration window, has been well established in primary sensory cortices in vitro and in vivo [1, 2, 29, 30]. However, physiological consequences of the broadened integration window could be drastically different. Considering that thalamofrontal excitatory synapses undergo rapid depression during high-frequency MD activity [24], frequency-dependent switching off of feedforward inhibition can be critical for continuous transfer of MD activity toward the PFC. In other words, during the maintenance of short-term memory, in which high-frequency MD activity must be transferred to the PFC [16], widening of the integration window can be critical for maintaining the activity loop between the MD and PFC.

Many uncertainties remain concerning the role of feedforward inhibition in the function of the PFC. In the sensory cortices, intra-cortical inhibitory synaptic transmission plays an important role in the construction of response selectivity. Upon inhibition, a significant reduction in the selectivity of neuronal responses to sensory stimuli such as orientation-selectivity and direction-selectivity has been reported [31–37]. Projection from MD drives disynaptic feedforward inhibition in the PFC as well [12], and blocking the feedforward inhibition leads to a significant alteration of the spatial selectivity of the neurons during working memory tasks [38]. However, in a considerable minority of PFC neurons, iontophoretically applied bicuculline unmasked new spatial tunings [38]. This result suggests that GABAergic transmission plays critical roles not only in the construction but also in switching of the response selectivity of PFC neurons. Although additional studies are required to test the impact of reduced feedforward inhibition on the spatial tuning of the PFC neurons, on/off switching of the response selectivity could occur during high-frequency MD-PFC activity, as seen in short-term memory. If this is the case, it is tempting to hypothesize that the switching of response selectivity by reduced feedforward inhibition might force different populations of neurons to activate at different time intervals during short-term memory tasks [39].

Fine regulation of the MD-mPFC connection strength is critical for normal function of the PFC. A subtle decrease in MD inputs reduced the functional synchrony of the MD and mPFC as well as cognitive functions [17, 40], and lesions of the MD recapitulated the cognitive impairments caused by PFC dysfunction, including loss of short-term memory [41, 42]. Altered functional connectivity between the MD and PFC has been reported in patients with short-term memory deficits [43, 44]. Furthermore, in patients with schizophrenia, decreased volume [45], number of neurons [46], and activity during short-term memory tasks have been observed in the MD [47]. Deteriorating alteration of feedforward inhibition in the PFC as a possible etiology of schizophrenia would be an interesting subject for future studies. Supporting this possibility, deficient output from PV neurons has been proposed [48] based on the decreased density of GABAergic neurons [49] without a decrease in the number of total neurons in cases of schizophrenia [50]. Altered transcriptional regulation of GABA_A_ receptor subtypes [51] and decreased expression of glutamate decarboxylase, which plays a role in the synthesis of GABA [52], in schizophrenic patients also support the possible engagement of feedforward inhibition.

Additional studies that directly measure the strength of feedforward inhibition during short-term memory tasks are required to examine the physiological significance of feedforward inhibition in the thalamofrontal circuit. In fact, the pattern of short-term depression on PV neurons can be assumed to be more moderate in vivo primarily due to ongoing neuronal activity and lower calcium concentration [53, 54]. The ongoing activity in vivo results in partially depressed synapses to begin with, and thus the short-term depression will be less pronounced compared to the initial amplitude [55, 56]. Moreover, the spontaneous network activity enhances the replenishment of the readily releasable pool of synaptic vesicles [57]. However, the reduced short-term depression due to ongoing activity is only a relative measure, and does not delay the depletion of a readily releasable pool. The MD-PV synapses in the mPFC have been demonstrated to be strongly depressed even in the presence of low calcium concentrations [24]. Furthermore, failure in excitation transfer occurred at as low as 5 Hz, which is a relatively low activity frequency compared to the firing rate observed in MD during the maintenance of short-term memory [17, 19, 20].

In summary, our study suggests that frequency-dependent on/off switching of feedforward inhibition serves as an active gating mechanism of the activity loop between the MD and mPFC, and thus finely controls the maintenance of short-term memory.

## Supplementary information

**Additional file 1: Supplementary Figure 1**. **A** Short-term plasticity of feedforward IPSCs on pyramidal cells (Black), PV-to-Pyramidal IPSCs (Green), and excitatory thalamofrontal EPSCs in pyramidal cells (Orange) at 5 Hz. No statistical difference was observed (unpaired *t*-test, parametric, *P* > 0.05), except the fifth response between MD-to-Pyr EPSC and Feedforward IPSC (See Fig. 3b also). **B** Short-term dynamics of the spike probability and EPSCs in PV cells by the 5 Hz MD stimulation (unpaired t-test, non-parametric, *P* > 0.05).

## Data Availability

The data used in our study are available from the authors on reasonable request.

## Abbreviations

AAV: Adeno-associated virus
aCSF: Artificial cerebrospinal fluid
AHP: After-hyperpolarization
AP: Action potential
APV: (2R) amino-5-phosphonopentanoate
ChR2: channelrhodopsin-2
CNQX: 6-cyano-7-nitroquinoxaline
dACC: Dorsal anterior cingulate cortex
EPSC: Excitatory postsynaptic current
IC_50_: Half maximal inhibitory concentration
IPSC: Inhibitory postsynaptic current
IW: Integration window
MD: Mediodorsal thalamic nucleus
mPFC: Medial prefrontal cortex
PSC: Postsynaptic current
PV: Parvalbumin
TTX: Tetrodotoxin
Vhold: Holding membrane potential
