# Supplementary figures and images for: Frequency-dependent gating of feedforward inhibition in thalamofrontal synapses
Source: Mol Brain. 2020 May 6;13:68. doi: 10.1186/s13041-020-00608-2 (PMC7201790; doi:10.1186/s13041-020-00608-2)

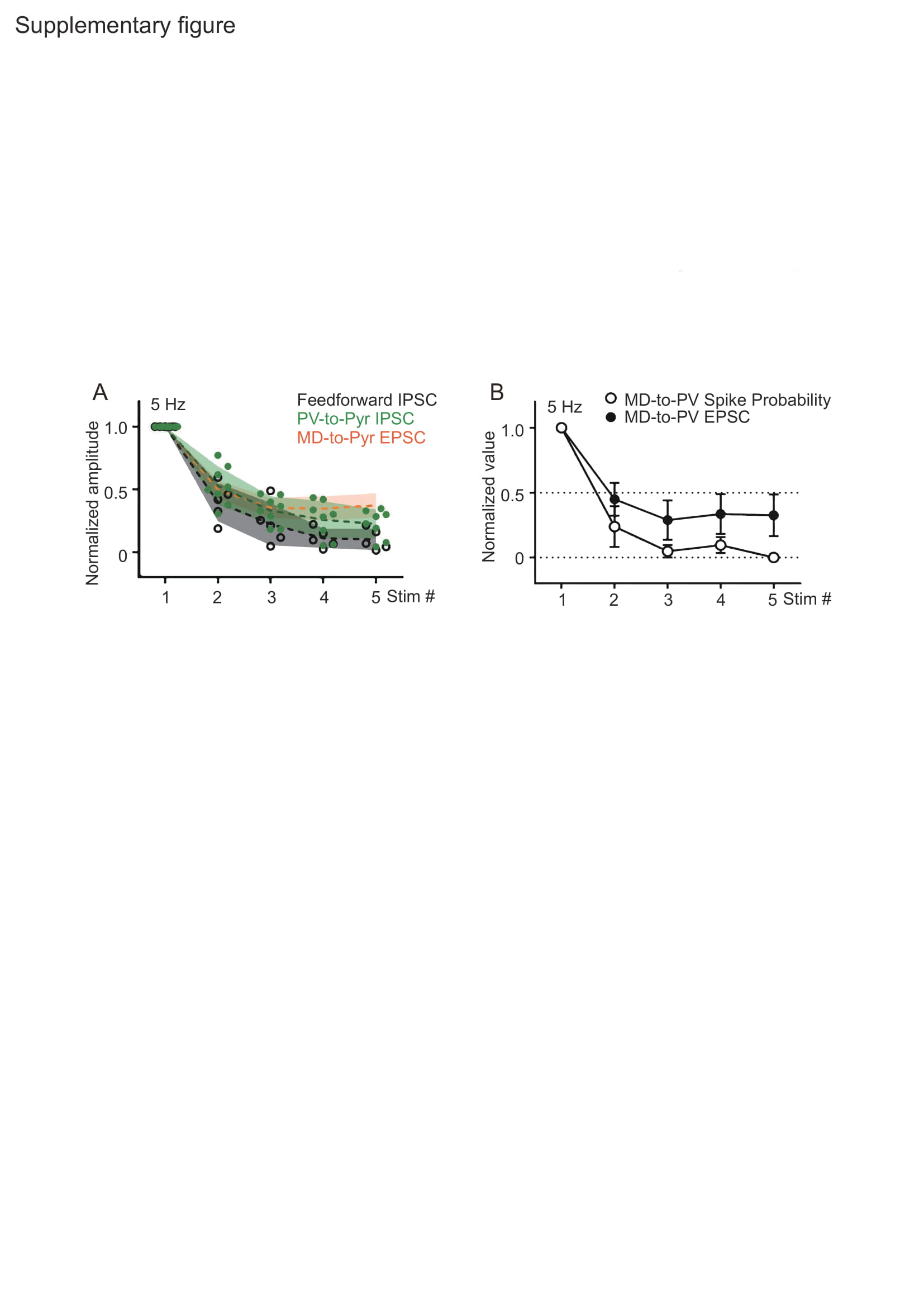

Supplement: Supplementary file 1 — Additional file 1: Supplementary Figure 1. A Short-term plasticity of feedforward IPSCs on pyramidal cells (Black), PV-to-Pyramidal IPSCs (Green), and excitatory thalamofrontal EPSCs in pyramidal cells (Orange) at 5 Hz. No statistical difference was observed (unpaired t-test, parametric, P > 0.05), except the fifth response between MD-to-Pyr EPSC and Feedforward IPSC (See Fig. 3b also). B Short-term dynamics of the spike probability and EPSCs in PV cells by the 5 Hz MD stimulation (unpaired t-test, non-parametric, P > 0.05). [file 13041_2020_608_MOESM1_ESM.jpg]
